# Polymyositis in Kooiker dogs is associated with a 39 kb deletion upstream of the canine *IL21/IL2* locus

**DOI:** 10.1371/journal.pgen.1011538

**Published:** 2025-01-02

**Authors:** Yvet Opmeer, Frank G. van Steenbeek, Claudia Rozendom, Hille Fieten, Montse M. Diaz Espineira, Qurine E. M. Stassen, Peter J. van Kooten, Victor P. M. G. Rutten, Marjo K. Hytönen, Hannes Lohi, Paul J. J. Mandigers, Peter A. Leegwater

**Affiliations:** 1 Expertise Centre of Genetics, Department Clinical Sciences, Faculty of Veterinary Medicine, Utrecht University, Utrecht, The Netherlands; 2 Section Immunology, Div Infectious Diseases and Immunology, Department Biomolecular Health Sciences, Faculty of Veterinary Medicine, Utrecht University, Utrecht, The Netherlands; 3 Dept of Veterinary Tropical Diseases, Fac of Veterinary Science, University of Pretoria, Republic of South Africa; 4 Department of Veterinary Biosciences, Department of Medical and Clinical Genetics, University of Helsinki and Folkhälsan Research Center, Helsinki, Finland; Wageningen University & Research, NETHERLANDS, KINGDOM OF THE

## Abstract

Recently we characterized polymyositis in the Dutch Kooiker dog. The familial occurrence of the disease were suggestive of an inherited cause. Here we report the results of our molecular genetic investigation. A genome-wide association study of 33 cases and 106 controls indicated the involvement of a region on chromosome CFA19 (p = 4.7*10^−10^). Haplotype analysis indicated that the cases shared a 2.9 Mb region in the homozygous or the heterozygous state. Next Generation Sequencing of genomic DNA implicated a deletion of a 39 kb DNA fragment, located 10 kb upstream of the neighbouring interleukin genes *IL21* and *IL2*. The frequency of the deletion allele was 0.81 in the available cases and 0.25 in a random sample of the Kooiker dog breed. Leukocytes of affected, untreated dogs that were homozygous for the deletion overexpress *IL21* and *IL2* upon stimulation with mitogens. We suggest that elements located 10–49 kb upstream of the *IL21*/*IL2* locus play an important role in the regulation of the canine genes and that deletion of these elements is a risk factor for polymyositis in Kooiker dogs. Postulating causality, the penetrance of the disease phenotype was estimated at 10–20% for homozygous dogs and 0.5–2% for dogs that were heterozygous for the deletion. Our results suggest that distant variants upstream of *IL21* could also be important for human autoimmune diseases that have been found to be associated with the *IL21*/*IL2* chromosome region.

## Author summary

Polymyositis in the breed of so-called Kooiker dogs is an autoimmune mediated inflammatory disease of skeletal muscles. The dogs are presented with swallowing and locomotion problems and in general the outcome is unfavorable. An inherited cause was suspected because affected dogs were often closely related. A genome-wide screen showed that the cases shared DNA variants in the region of the interleukin genes *IL21* and *IL2*, wich are regulators of the cellular immune response. By analysis of the DNA sequence it became evident that a fragment of 39,000 base pairs had been deleted from the shared region. The deletion is located in front of the *IL21* gene. All but two affected dogs had one or two copies of the deletion. Kooiker dogs with two copies have a risk of 10–20% to develop polymyositis in their lifetime; for dogs with one copy the risk is only 0.5–2%. We suggest that the deletion disturbs the normal regulation of *IL21* and/or *IL2* expression. Our results could be important for the research of human autoimmune diseases that have been associated with the same gene region.

## Introduction

An autoimmune-mediated inflammatory myopathy (IM)was seen with increasing frequency in the breed of Nederlandse Kooikerhondjes, named Kooiker dogs hereafter [1]. The dogs presented with locomotion problems with or without dysphagia. Serum creatine kinase activity was almost invariably elevated, and histopathology of available muscle biopsies revealed predominantly lymphohistiocytic infiltration usually showing a low and variable number of eosinophils, neutrophils and plasma cells [1]. Detailed pathological investigation and immunological characterization of cellular infiltrates were indicative of polymyositis (PM) [2]. The condition occurred more often in the Kooiker dog breed than in other breeds and littermates of affected dogs appeared to be at increased risk for developing PM. Therefore, a genetic background was suspected. Dog breeds in general have been established with a small number of founders, are therefore genetically homogeneous and excellent sources for molecular research of monogenic and polygenic traits [3–6].

Inherited causes of distinct forms of IM have been investigated in Vizsla dogs, Boxers, Newfoundland dogs, Dutch shepherd dogs, Shetland sheepdogs and Collies [7–10]. One haplotype of MHC class II genes appeared to be a risk factor for PM in Vizsla dogs [7]. Another MHC class II haplotype was associated with dermatomyositis in the related breeds of Shetland sheepdogs and Collies [8]. Additional risk variants in these two breeds were recognized in the genes *PAN2* and *MAP3K7CL*, and the risk of dermatomyositis increased with the total number of risk alleles carried by the dogs. An autosomal recessive IM in Dutch shepherd dogs in an isolated breeding line in the US was reported to be caused by a variant in the mitochondrial aspartate and glutamate carrier encoded by *SLC25A12* [9]. The homozygous mutant genotype appeared to lead to the IM phenotype with complete penetrance.

Autoimmune diseases in humans have frequently been associated with specific alleles or haplotypes of the HLA locus. Indeed, the strongest genetic risk factor for idiopathic IM in humans is located in the HLA region [11]. Other genes that have been associated with human idiopathic IM and PM include *SDK2*, *LINC00924*, *STAT4*, *NAB1*, *TEC*, *LTBR*, *DGQK*, and *FAM167A-BLK* [11].

At the functional level, it was shown that interleukin 21 (IL-21) is expressed by cellular infiltrates in muscles of PM patients [12]. The immune system depends largely on the communication between leukocytes through a variety of interleukins. Unraveling the complex network of interleukin expression and their actions is a major effort in the research of the immune system and autoimmune diseases. We set out to study the molecular genetics of the specific form of PM that is prevalent in the Kooiker dog breed.

## Material and methods

### Ethics statement

The dogs entered in this study were examined and handled by licensed veterinarians. Tests were performed and samples were collected in the course of routine diagnosis and care. All dogs were privately owned and included with informed consent of their owners. Thus we complied to the conditions set forth in the Dutch ‘Wet op de Uitoefening van de Diergeneeskunde’ (Law on the Practice of Veterinary Medicine) of March 21, 1990 and approval of an ethics committee for the use of samples of the animals was not necessary.

### Dogs

Affected purebred Kooiker dogs were referred to the veterinary neurology unit of the University Clinic for Companion Animals of Utrecht University by owners, made known to us by veterinary practitioners, or retrieved from the breed club register of the VHNK (Association the Nederlandse Kooikerhondje). The cases fulfilled at least three of the four criteria of Bohan and Peter [13]: 1) symmetric proximal muscle weakness; 2) elevated muscle enzymes; 3) spontaneous activity on the EMG; 4) characteristic muscle biopsy abnormalities as described [1]. The DNA was isolated from EDTA-blood by use of a Chemagen MSM I robot or Magcore HF16plus robot using consumables and protocols supplied by the manufacturers. The DNA of 102 affected dogs was available. The unaffected controls were Kooiker dogs of which the DNA was tested earlier at the Expertise Centre of Genetics, Faculty of Veterinary Medicine, Utrecht University for gene variants involved in Von Willebrand disease or hereditary necrotizing myelopathy [3,4]. The DNA of more than 4,000 Kooiker dogs born between 1988 and 2022 was available. Parents and ancestors of cases were retrieved using pedigree data. A group of 112 dogs was compiled to serve as a random comparison group for allele frequency measurement.

Survival times and box plots in relation to genotypes were analyzed with SPSS version 29.

### Molecular genetics

An initial group of 28 Kooiker dog PM cases was sent for genotyping of 172,115 single nucleotide polymorphisms (SNPs) of the Illumina CanineHD BeadChip to the company Neogen. The control group of 106 Kooiker dogs was genotyped on the same platform for unrelated projects at the Centre National de Génotypage, Paris, France. The case-control comparison was performed with Genabel software version 1.6–6 with adjustment for stratification and relatedness as described [14]. Haplotypes were deduced using PHASE 2.1 [15]. Homozygosity mapping was done with PLINK software version 1.9 using default settings [16].

Fragments of all gene exons and adjacent 25 bp in the region of interest on CFA19 from position 15,000,000–24,500,000 plus the complete upstream, introns and intergenic fragments of the *IL21* and *IL2* genes from position 17,554,000–17,757,000 within that region were enriched with Agilent Sureselect technology from sheared DNA of 8 PM affected and 4 unaffected Kooiker dogs. The genes of which the exons were included in the enrichment are listed in S1 Table. Library preparation was performed using the Illumina TruSeq Nano DNA Prep Kit. The enriched libraries were sequenced on an Illumina MiSeq with a coverage of 100x on average. Mapping and variant calling of the paired-end reads of 150 bases were achieved using a custom bioinformatic pipeline based on the Burrows–Wheeler Aligner algorithm [17]. SAMtools was used for annotation and initial variant calling against the reference genome CanFam3.1 release 75 [18]. The positions of the variations were adjusted to the reference genome UU_Cfam_GSD_1.0 using a liftover tool [19]. The possibly functional effect of DNA sequence variants was evaluated with snpSift and snpEff version 4.1 [20]. In addition, the region of the *IL21*/*IL2* locus was inspected for rearrangements using Integrative Genome viewer version 2.5.0 [21]. Later, three genomes, one of a healthy Kooiker dog that was homozygous for the identified risk allele and sired an PM case, and two heterozygous cases, were fully sequenced with a coverage of 30x and mapped to UU_Cfam_GSD_1.0 using methods described above. The DNA sequence data of these dogs has been made available (SRA BioProject accession number PRJNA1092409).

To evaluate the presence of a 39 kb deletion in Kooiker dogs, a PCR was designed with a single forward primer I (CTCCAGAGAAGAACCACCACT), a regular reverse primer II (TGTGGTTTGAATTTTGCATCAGG) located in the deleted fragment, and a deletion reverse primer III (TCACATGTGCCACAAATAAGGT) that spans the 39 kb fragment. PCR using primer pair I/II produces a fragment of 662 bp from the reference allele only, while pair I/III yields a product of 242 bp that is specific for the deletion allele. The PCR was performed with Platinum Taq, using standard conditions and an annealing temperature of 55°C. Products were separated and visualized by electrophoresis on a 1.8% agarose gel containing ethidium bromide.

The LOD score for linkage between the inheritance of the phenotype and the deletion was calculated for an extended pedigree with Superlink [22].

### *IL21*/*IL2* expression studies

Peripheral blood mononuclear cells (PBMCs) were isolated from 8 ml of heparinized whole blood of two cases and one control. Whole blood was mixed with an equal volume of RPMI 1640 medium. The diluted blood was layered on a Histopaque 1077 density gradient and centrifuged for 20 minutes at 800x g at 20°C. The interphase layer with the PBMCs was recovered, adjusted to 50 ml with RPMI 1640 medium in a new sterile 50 ml tube, and centrifuged for 7 minutes at 400x g at 20°C. The supernatant was discarded and the cell pellet was resuspended in RPMI 1640 medium supplemented with gentamicin and 5% fetal calf serum, and the cells were counted. The cell suspensions were diluted to 4*10^6^ cells/ml. Cells were seeded in flat-bottomed 6-well plates, 12*10^6^ cells in 3 ml per well, in duplicate. For stimulation, the cells were incubated with 50 ng/ml phorbol myristate acetate and 1 μg/ml Ca-ionophore for 24h at 37°C, at 7% CO_2_. Control incubations were performed without the stimulating agents. Cells were harvested by centrifugation for 7 min at 250x g, supernatant was discarded and the cell pellets stored at -20°C until further processing.

Total RNA was extracted from PBMCs using the RNeasy Mini Kit (Qiagen) according to the manufacturer’s instructions and further purified by incubation with RNase-free DNase I (Qiagen). The isolated RNA was quantified by NanoDrop. The RNA was transcribed into cDNA by the I-script cDNA synthesis kit (BioRad) according to the manufacturer’s instructions and the cDNA was stored at -20°C.

Quantitative PCR was performed with SYBR green using standard conditions. The primers for the *IL2* gene were from an earlier study [23] and the primers for *IL21* were designed using primer3 software [24]. The genes *RPS19*, *HNRPH* and *SDHA* were used as references genes. Details of the primers are listed in S2 Table.

The PCR was performed with a 3-step protocol on a qPCR Biorad iQ5: initial denaturation at 95°C for 5 min, followed by 50 cycles of 95°C for 20 s, annealing temperature for 20 s and 72°C for 20 s; and finally, a melt curve step from 60°C to 98°C. Each plate had an internal standard of pooled cDNA from all samples and all samples were tested in duplicate. All the samples were normalized to the geometric mean of the reference genes.

## Results

### An PM locus on chromosome 19

**Fig 1:**
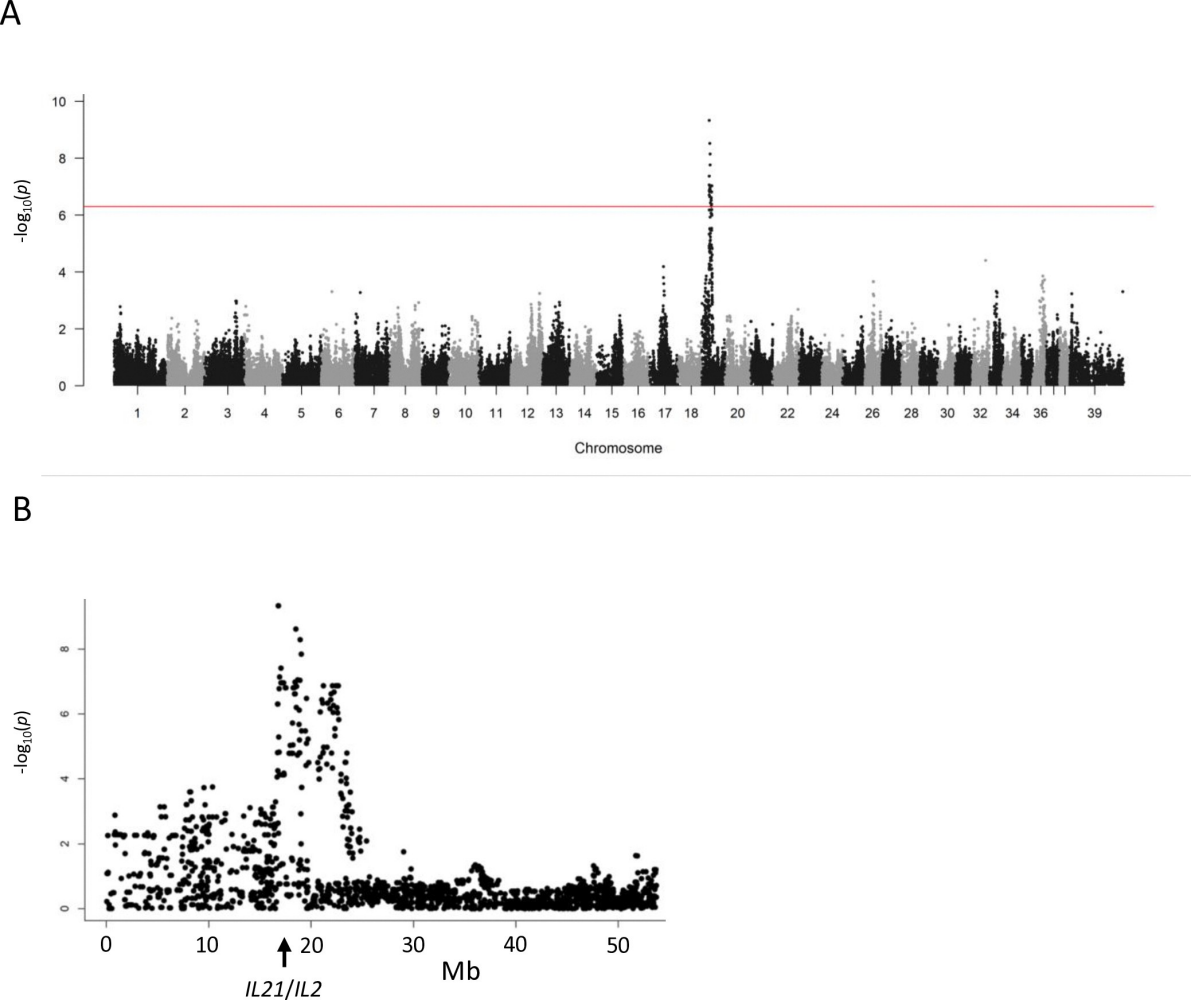
Genome-wide association of polymyositis in Kooiker dogs. Twenty-eight affected dogs and 106 controls were genotyped at more than 170,000 SNP positions. Differences between the groups were evaluated with Genabel software and the -log_10_(*p*) value is plotted against the chromosomal position. A) Manhattan plot summarizing results from all chromosomes. The red line marks the significance threshold after Bonferroni correction. B) Results from chromosome 19. The position of the *IL21*/*IL2* locus is indicated.

The genome-wide association study resulted in a single highly significant signal on CFA19 (Fig 1). The top SNP was BICF2P1435772 at UU_Cfam_GSD_1.0 position NC_049240.1:g. 17605165 = with a p-value of 4.7*10^−10^. Haplotype construction with the data of the top 3 informative SNPs suggested that 19 cases were homozygous for the same haplotype while the remaining 9 cases each had one copy of this haplotype (S3 Table). Of the 106 controls, 5 and 37 dogs were homozygous and heterozygous for the haplotype, respectively. Since all cases had one or two copies of the haplotype and the same genotypes were also represented in the control group, we suggested that the mode of inheritance of PM was semidominant with incomplete penetrance. A majority of the cases shared the same haplotype homozygously; therefore, the critical region was defined by homozygosity mapping (S4 Table). Twenty cases had overlapping runs of homozygosity in the CFA19 region of interest. Multiple cases supported the localization of the critical region at canfam3.1 positions CM000019:g.16624866_19515741 = (UU_Cfam_GSD_1.0 annotation NC_049240.1:g.17443487_20354727 =).

None of the genes involved in idiopathic IM, PM, or dermatomyositis in other dog breeds was situated in the region. The region did contain the cytokine genes *IL21* and *IL2*, which encode regulators of cell-mediated immunity. Targeted resequencing was performed of all coding regions and splice sites across the critical chromosome fragment plus the complete 88 kb upstream region, introns and intergenic region of *IL21* and *IL2* of 8 cases and 4 control Kooiker dogs. The DNA sequences did not reveal protein-coding variants that warranted further investigation of their possible role in the phenotype because these were not associated with the phenotype in the sequenced sample (S5 Table).

**Fig 2:**
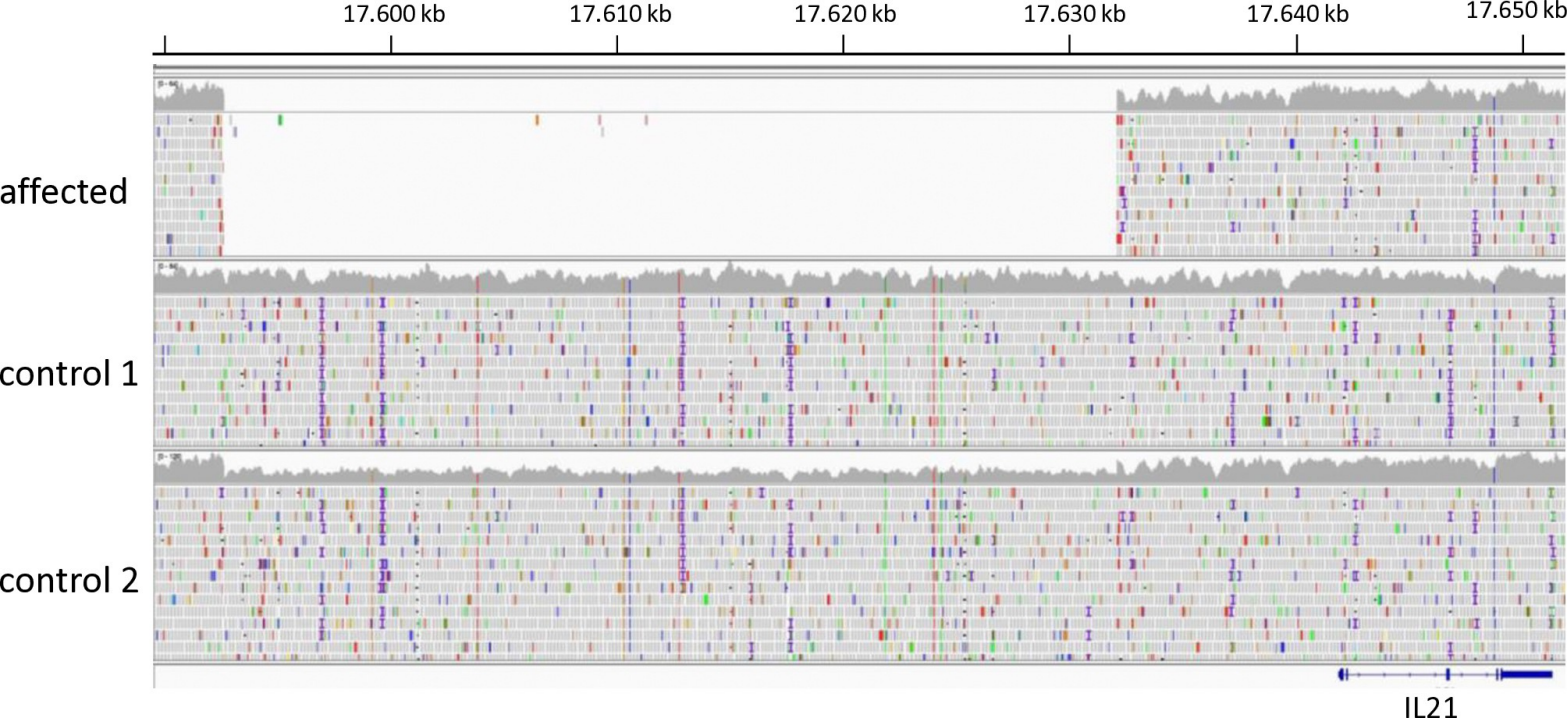
Integrative genomics view of next generation sequence data of the region upstream of *IL21* in Kooiker dogs. The DNA of the polymyositis affected dog did not generate data from a 39 kb region. The same was observed in the data of the other 7 affected dogs that were analyzed by NGS. Control 1 shows average coverage in the region, while the coverage of control 2 is approximately half of that. A PCR assay, which distinguishes the reference and the deletion allele confirmed the heterozygosity of this control. The position on CFA19 corresponds to CanFam3.1. The corresponding positions of the borders of the deletion on the reference genome UU_Cfam_GSD_1.0 are 18,414,090 and 18,453,526.

We then postulated that regulatory elements of gene expression could be involved in the pathogenesis. Inspection of the resequenced region with IGV showed that all 8 affected dogs were homozygous for a deletion of 39 kb, located 10–49 kb upstream of *IL21* (Fig 2). The deletion stretched from CFA19 positions 18,414,090–18,453,526 (UU_Cfam_GSD_1.0 annotation NC_049240.1:g.18414090_18453526del =). Judging from the read coverage of the 39 kb region, two of the controls were heterozygous for the deletion. The deletion fragment did not contain nor overlapped with known protein-coding genes. The later generated whole genome sequences of three Kooiker dogs, one of which was homozygous for the deletion allele and two that were heterozygous, did not reveal variants in the critical region that justified further investigation (S6 Table). All variants in the region with a predicted high or moderate effect on protein function were represented in the variant database dog10k derived from genome sequences of 1987 dogs with substantial frequencies [25]. Low impact variants that appeared to be in complete LD with the 39 kb deletion in the three dogs were also represented in dog10k. We concluded that the 39 kb deletion was the most likely and only risk factor for the development of PM in the CFA19 region, i.e. without allelic heterogeneity.

**Fig 3:**
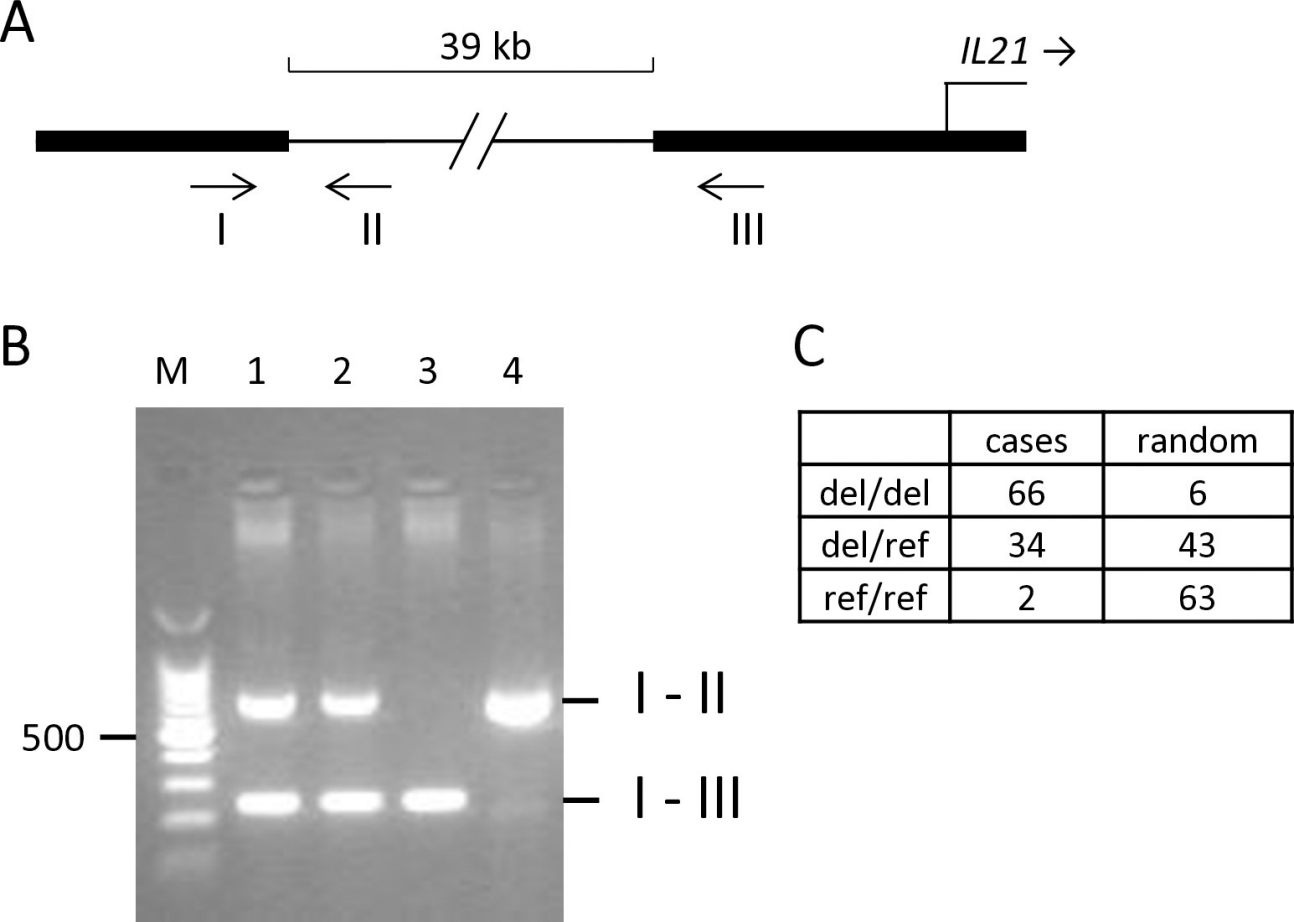
Three primer assay for genotyping of the 39 kb deletion variant upstream of *IL21*. A) The positions of the PCR primers I, II and III are indicated relative to the 39 kb deletion. The combination I–II generates a product of 662 bp from the normal variant. The combination I–III produces a fragment of 242 bp from the deletion variant only. B) Result of the assay for heterozygotes (lanes 1 and 2), a dog homozygous for the deletion (lane 3), and a homozygous normal dog (lane 4). C) Genotype distribution of the 39 kb deletion (del) in 102 IM cases and 112 random Kooiker dogs.

A PCR assay with three primers was developed that distinguished the deletion allele from the reference allele by the length of the respective products (Fig 3). Of the available cases, 66 were homozygous for the deletion, 34 were heterozygous and two were clear of the deletion. The frequency of the deletion allele was 0.81 in the cases and 0.25 in a random group of Kooiker dogs. The assay confirmed that two of the controls included in the NGS analysis were heterozygous. The genotype distribution (Fig 3C) of the cases differed from that of the random group with a chi-square test p-value of 5*10^−157^.

**Fig 4:**
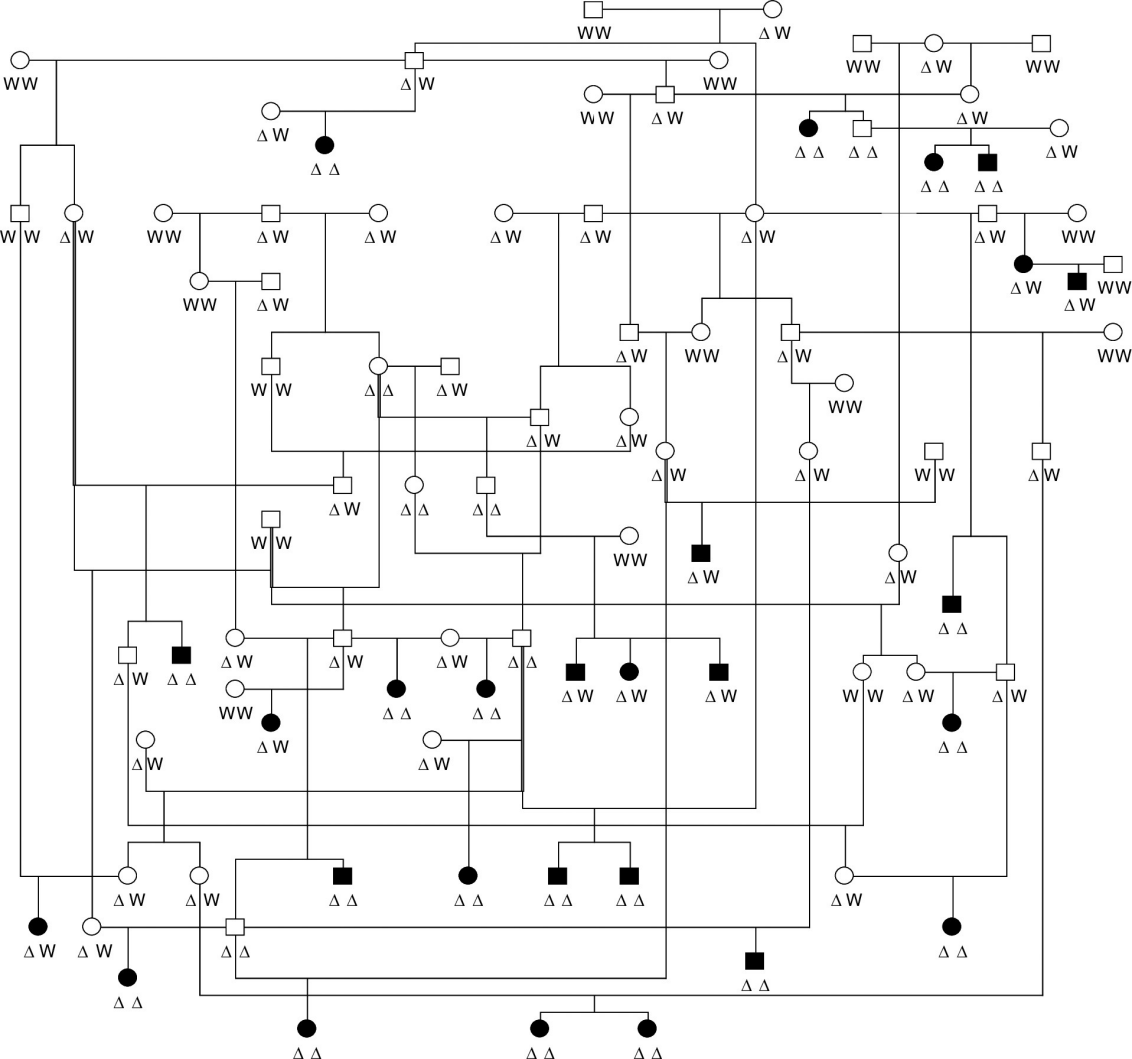
Segregation of the 39 kb deletion and polymyositis in a pedigree of Kooiker dogs. The birth year of the first-born founder of the pedigree was 1992 and DNA of all dogs was available. W: reference allele; Δ: 39 kb deletion allele. The frequency of the deletion allele was 0.24 in the 29 founders of this pedigree and 0.85 in the 27 affected dogs.

The deletion segregated with PM in a pedigree of 5 generations with 89 Kooiker dogs including 27 cases (Fig 4). The LOD score for linkage between the phenotype and the deletion in this pedigree was 4.7 at recombination fraction (Θ) 0, with the penetrance for heterozygotes set at 0.01 and for dogs homozygous for the deletion allele at 0.1.

### Genotype-phenotype relationship

We evaluated whether the homozygous or heterozygous state of the deletion allele was related to the age at first presentation or the survival time after diagnosis. The homozygous cases had a mean age of onset of 3.73 years with a standard deviation of 2.02 years. The mean age of onset of the heterozygous cases was more than a year higher at 5.05 ± 2.33 years (S1 Fig). The difference between the groups was significant with a p-value of 0.05. The Kaplan-Meier curves did not show a significant difference between the survival time after diagnosis between homozygous and heterozygous dogs (S2 Fig).

### Expression of *IL21* and *IL2* in PBMCs

**Fig 5:**
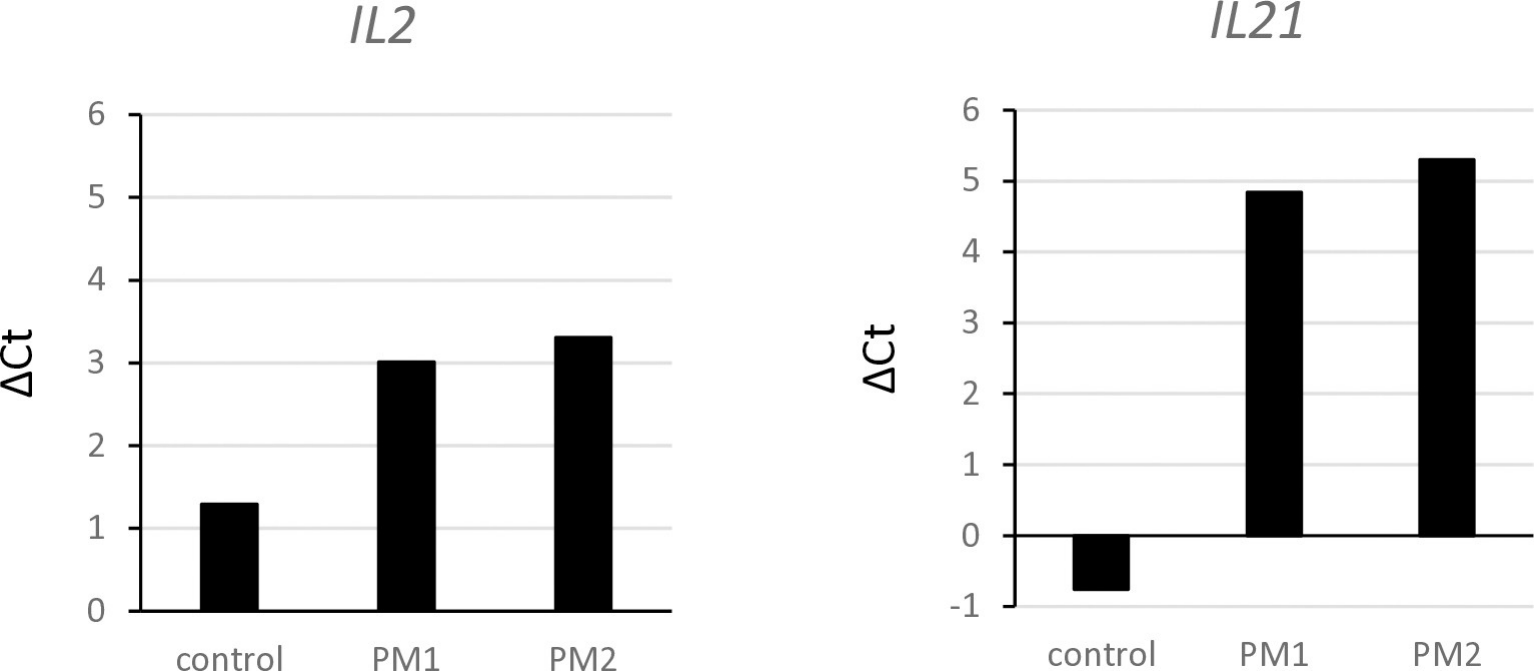
Stimulation of *IL21* and *IL2* expression in PBMCs from PM affected dogs. Blood samples for leukocyte isolation were taken from two untreated Kooiker dogs with PM. The dogs PM1 and PM2 were homozygous for the 39 kb deletion. The cells were cultured with and without stimulating mitogen, RNA was isolated and RT-qPCR performed. Plotted are the Ct-values of the unstimulated cells minus the Ct-values of the stimulated cells from the same dogs. The control cells were left over from a routine diagnostic procedure. The control dog did not have the 39 kb deletion.

We imagined that the 39 kb fragment situated close to *IL21* contains elements that influence the expression of this gene and possibly the neighboring *IL2* gene. In a preliminary experiment, cells from a control dog that was clear of the 39 kb deletion displayed small differences in expression of the two interleukin genes between the stimulated and the unstimulated state (Fig 5). The PBMCs of two affected Kooiker dogs, which were isolated before treatment of PM, displayed substantial increases in expression of *IL21* as well as *IL2* by the stimulation. Both affected dogs were homozygous for the 39 kb deletion. These results suggest that the deleted region contains elements that affect the regulation of the interleukin genes.

## Discussion

Kooiker dogs presented an autoimmune myopathy that we could analyze genetically because of the homogeneous makeup of the breed population. We linked the disease to a deletion of a DNA region of 39 kb, upstream of two interleukin genes. The obtained LOD score is well above the threshold value of 3.0, which is conventionally considered proof of linkage. The score of 4.7 means that the likelihood that the phenotype is linked to the DNA variant is 10^4.7^ = 50,000 times larger than the likelihood that they are not linked. The deleted region does not contain protein-coding exons according to the recent genome annotation (UU_Cfam_GSD_1.0). We postulated that the deletion affects the regulation of one or both of the adjacent interleukin genes *IL21* and *IL2*, thereby increasing the risk for superfluous inflammatory reactions. According to data in TADKB [26], the two genes are located on the same topologically associating domains (TADs) in diverse human cell lines, suggesting their expression could be coordinated. Indeed, the expression of both genes is enhanced strongly in PBMCs from untreated PM affected Kooiker dogs that are homozygous for the deletion. Leukocytes from a control dog that did not have the deletion showed little change of expression of the genes. The expression study needs to be replicated in a larger cohort to confirm the effect of the 39 kb deletion on the regulation of the genes.

Interestingly, a similarly sized deletion upstream of *IRGM* in humans has been associated with inflammatory bowel disease 19 (Crohn disease 19) [27]. The deletion of 20 kb at a distance of 2,7 kb from *IRGM* leads to up- or downregulation of the gene, depending on the cell type.

The mode of inheritance of Kooiker dog PM seems to be semidominant with incomplete penetrance. Of the cases, 65% is homozygous for the deletion and 33% is heterozygous. Homozygotes tend to develop the disease at a younger age than heterozygotes. With approximately 15,000 Kooiker dogs from which the cases were recruited, and a deletion allele frequency of 0.25, we estimate that the penetrance of PM is between 0.1 and 0.2 for homozygous dogs and between 0.005 and 0.02 for heterozygous dogs, depending on the report level of all cases to the breed club. It is very well possible that other inherited factors modify the risk of PM in Kooiker dogs.

Only two of 102 available Kooiker dogs with PM do not have the deletion. These two dogs have the same mother while their fathers are first cousins. Possibly these two cases are phenocopies with a completely different pathogenesis. It is also possible that the two isolated cases share yet unknown risk factors with other cases.

The role of IL-21 in autoimmunity has been studied extensively (reviewed in [28]). Mainly expressed by activated CD4^+^ T cells, it promotes the proliferation of natural killer cells and cytotoxic T cells. Overexpression of IL-21 is observed in tissues and sera of human patients with autoimmune diseases such as systematic lupus erythematosus (SLE), rheumatoid arthritis (RA), type 1 diabetes mellitus (DM), and primary Sjögren syndrome (SS) [28,29]. Specifically, expression of the *IL21* gene was found to be upregulated in patients with PM and dermatomyositis [12]. Clearly, enhanced expression of *IL21* can be instrumental in the etiology of these autoimmune diseases.

The role of the interleukin encoded by the neighboring gene *IL2* in autoimmunity seems to be the opposite of that of IL-21. Not overexpression but *Il2* knockout leads to a disrupted immune response and predisposition to inflammatory bowel disease [30,31]. The interleukin IL-2 has been investigated as a therapeutic agent in autoimmune and inflammatory diseases with promising results [32]. We conclude that dysregulation of *IL21* expression is the most likely cause of PM in Kooiker dogs. Follow-up experiments should be directed to fine-mapping of the DNA elements in the 39 kb fragment that are involved in the expression of *IL21*. By targeted deletion of smaller DNA fragments in a wild-type cell line, capable of *IL21* expression in response to stimulation, these elements could be identified.

Genome-wide association studies established a possible role of the *IL21*/*IL2* region in the human autoimmune diseases RA, SLE, type 1 DM, and SS [33]. No DNA variants have been identified that are instrumental in the risk of the diseases. Our results indicate that these variants may be found at a distance of at least 10 kb upstream of the *IL21* gene.

Our study unequivocally demonstrates the involvement of the *IL21*/*IL2* locus in polymyositis in the Kooiker dog. However, the responsible DNA element and its function in inflammatory processes are yet to be discovered. It will be interesting to learn whether human cases of autoimmune diseases that map to the *IL21*/*IL2* locus carry specific variants in the region syntenic to the 39 kb deletion. Meanwhile, the PCR assay to genotype the deletion allele in Kooiker dogs provides a tool for breeders to prevent the birth of high risk dogs and reduce the incidence of polymyositis in the breed.

## Supporting information

S1 Fig Comparison of the age of onset of polymyositis in Kooiker dogs by genotype. The boxplots are based on 59 cases homozygous for the 39 kb deletion (-/-) and 25 heterozygous cases (-/+). The mean age of onset of -/- dogs was 3.73 ± 2.02 yrs and of -/+ dogs 5.05 ± 2.33 yrs (t-test p = 0,05).
(TIF)

S2 Fig Kaplan-Meier curves for survival of Kooiker dogs after diagnosis of polymyositis. The blue line indicates the group of dogs who were homozygous for the 39 kb deletion (59 cases), the red line those who were heterozygous (25 cases). The homozygous cases had a survival time of 14.7 ± 16.7 months, the heterozygous cases 14.4 ± 16.9 months (t-test p = 0.47).
(TIF)

S1 Table Genes of which exon fragments were enriched for Next Generation Sequencing. (XLSX)

S2 Table Oligonucleotide sequences used in qPCR. (XLSX)

S3 Table Haplotypes of the CFA19 region of interest in PM cases and controls of the GWAS. (XLSX)

S4 Table Homozygosity mapping of the CFA19 region associated with PM in Kooiker dogs. (XLSX)

S5 Table SnpEff output of next generation sequence data of the chromosome 19 region of interest from 8 cases and 4 controls. (XLSX)

S6 Table Evaluation WGS data, limited to the CFA19 region critical for PM in Kooiker dogs. (XLSX)
