# Supplementary figures and images for: Polymyositis in Kooiker dogs is associated with a 39 kb deletion upstream of the canine IL21/IL2 locus
Source: PLoS Genet. 2025 Jan 2;21(1):e1011538. doi: 10.1371/journal.pgen.1011538 (PMC11731761; doi:10.1371/journal.pgen.1011538)

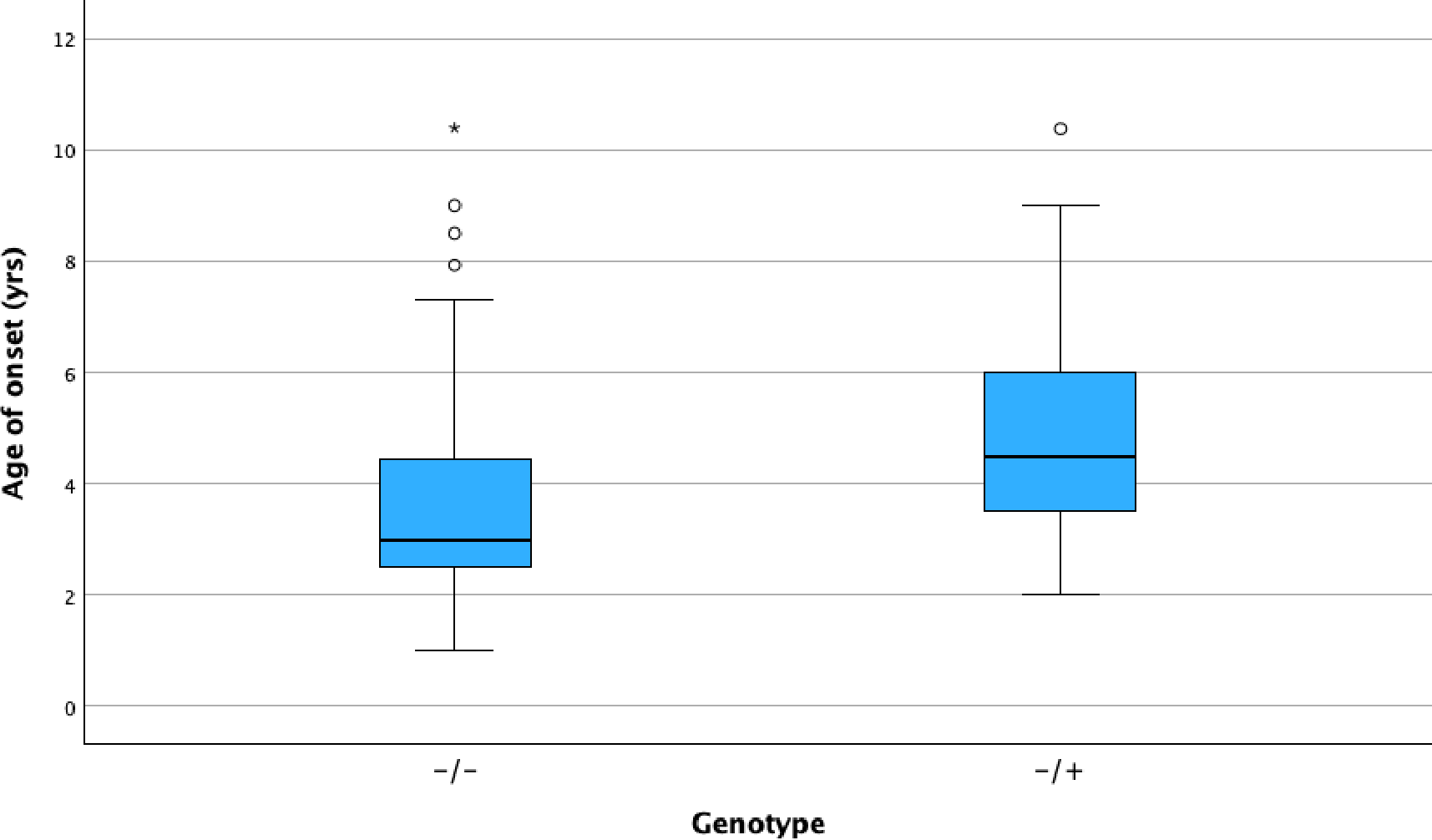

Supplement: S1 Fig — The boxplots are based on 59 cases homozygous for the 39 kb deletion (-/-) and 25 heterozygous cases (-/+). The mean age of onset of -/- dogs was 3.73 ± 2.02 yrs and of -/+ dogs 5.05 ± 2.33 yrs (t-test p = 0,05). (TIF) [file pgen.1011538.s001.tif]

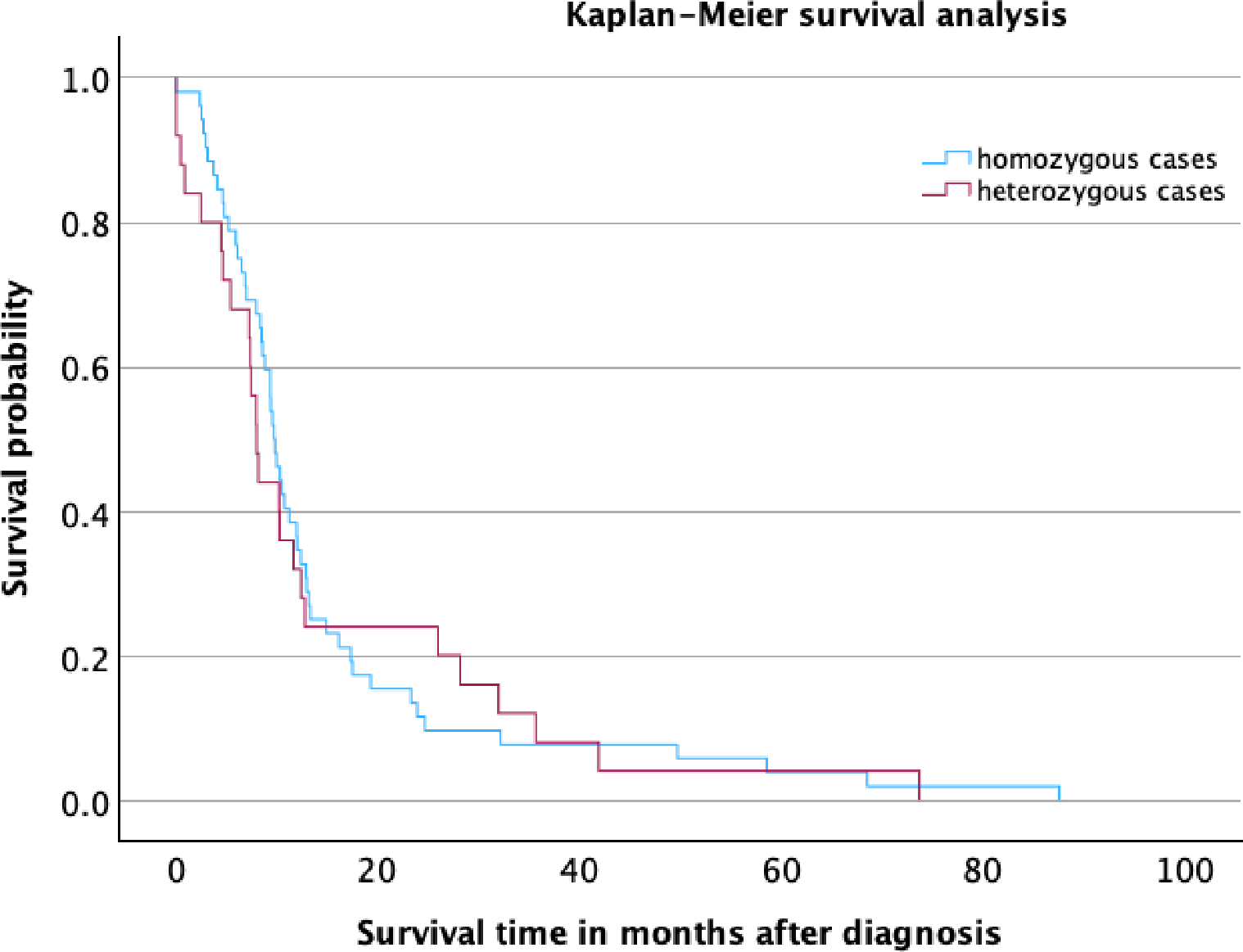

Supplement: S2 Fig — The blue line indicates the group of dogs who were homozygous for the 39 kb deletion (59 cases), the red line those who were heterozygous (25 cases). The homozygous cases had a survival time of 14.7 ± 16.7 months, the heterozygous cases 14.4 ± 16.9 months (t-test p = 0.47). (TIF) [file pgen.1011538.s002.tif]
